# Unequal geographic distribution of water and sanitation at the household and school level in Sudan

**DOI:** 10.1371/journal.pone.0258418

**Published:** 2021-10-15

**Authors:** Seungman Cha, Yan Jin, Mousab Siddig Elhag, Youngjin Kim, Hassan Ahmed Hassan Ahmed Ismail

**Affiliations:** 1 Department of Disease Control, London School of Hygiene & Tropical Medicine, London, United Kingdom; 2 Department of Global Development and Entrepreneurship, Graduate School of Global Development and Entrepreneurship, Handong Global University, Pohang, South Korea; 3 Department of Microbiology, Dongguk University College of Medicine, Gyeongju, South Korea; 4 Communicable and Non-Communicable Diseases Control Directorate, Federal Ministry of Health, Khartoum, Sudan; National University of Sciences and Technology, PAKISTAN

## Abstract

The Sudanese Government launched the National SDG-6 Plan and commences its implementation to achieve and sustain universal and equitable access to basic WASH services by 2030. It is critical to understand the geographical heterogeneity of Sudan and patterns in the inequality of access to safe drinking water and sanitation. Through such research, the disease control strategy can be optimized, and resource allocation can be prioritized. We explored spatial heterogeneity and inequality in access to improved water and sanitation across Sudan by mapping the coverage at both the state and district levels. We decomposed the inequality across Sudan into within-state, between-state, within-district, and between-state inequalities using the Theil L and Theil T indices. We calculated the Gini coefficient to assess the inequality of access to improved water and sanitation, based on the deviation of the Lorenz curve from the line of perfect equality. The study population was 105,167 students aged 8–13 at 1,776 primary schools across the country. Geographical heterogeneity was prominent in the Central Darfur, South Darfur, East Darfur, Kassala, West Kordofan, and Blue Nile States, all of which showed severe inequality in access to an improved latrine at the household level in terms of the Theil T or Theil L index. The overall inequality in the coverage of improved sanitation went beyond the warning limit of 0.4 for the Gini coefficient. The inequality in terms of the Theil L and Theil T indices, as well as the Gini coefficient, was always higher for improved sanitation than for improved water at the household level. Within-state inequality accounted for 66% or more of national inequalities in the distribution of improved sanitation and drinking water for both the Theil L and Theil T indices. This is the first study to measure geographical heterogeneity and inequalities in improved water and sanitation coverage across Sudan. The study may help to prioritize resource allocation to areas with the greatest water and sanitation needs.

## Introduction

Safe drinking water and sanitation are associated with reduced susceptibility to waterborne diseases and many neglected tropical diseases. As such, access to an improved water supply and sanitation is a basic human right and an essential component of human dignity [1]. The Joint Monitoring Programme for Water Supply and Sanitation (JMP) has emphasized the importance of sanitation facilities that hygienically isolate human excreta from human contact and drinking-water sources that guard against contamination [2]. Globally, a total of 2 billion people are still living without access to sanitation facilities in 2019 [2]. Many studies have suggested that inequalities of water and sanitation coverage exist among socioeconomic groups, with an unequal distribution across geographic locations within a country [3–9]. However, to our knowledge, no studies have estimated the geographical distribution of water and sanitation in Sudan.

The JMP reported that the coverage of at least basic sanitation in Sudan reached 37% in 2017, while 24% of the population was still reported to be practicing open defecation [2]. Diarrhea was responsible for 537,492 DALYs (95% UI 254,321–1,020,827) among Sudanese children younger than 5 years in 2016 [10]. Having an unprotected water source and poor sanitation have been reported to be major contributors to the burden of diarrheal disease in Sudan, as well as in other countries [11].

The Sudanese Government launched the National SDG-6 Plan and commences its implementation to achieve and sustain universal and equitable access to basic WASH services by 2030 [12]. In addition, UNICEF Sudan, together with the Sudanese government, set the goal of making Sudan open-defecation-free by 2022 [13]. In a bid for these goals to be achieved, it is critical to understand the geographical heterogeneity of Sudan and patterns in the inequality of access to safe drinking water and sanitation. Through such research, the disease control strategy can be optimized, and resource allocation can be prioritized.

We aimed to explore spatial heterogeneity and inequality in access to improved water and sanitation across Sudan by mapping the coverage at both the state and district levels. Inequalities were assessed within and between these administrative levels, and the specific states or districts in greatest need in terms of water and sanitation coverage were identified to draw policy attention.

## Methods

A nationwide water, sanitation, and hygiene (WASH) survey was carried out under the umbrella of the SUKO project, which was the Sudan and Korea Collaboration Project of Schistosomiasis and other Intestinal Helminthiases Control, supported by the Korea International Cooperation Agency (KOICA). The survey protocol has been described previously [14].

### Study area

Sudan is the third largest country in Africa, comprising 189 districts in 18 states. From the highest level to the lowest, the administrative divisions are states, districts, and administrative units [15]. The estimated Sudanese population was 37.4 million in 2016, of whom 45.6% were children younger than 15 years and 3.9% were aged above 59 years. The White Nile, the Blue Nile, and the Nile River flow through the country. The study population was students aged 8–13 at 1,772 primary schools across the country.

For MDG monitoring, an improved sanitation facility was described as “hygienically separating human excreta from human contact,” while an improved drinking-water source was defined as being “protected from outside contamination (especially fecal contamination)” [16].

The JMP defines four types of facilities as improved: (1) flush or pour-flush to piped sewer system/septic tank/pit latrines; (2) ventilated improved pit (VIP) latrines; (3) pit latrines with a slab; and (4) composting toilets. It also defines six types of improved drinking-water sources: (1) piped water into the dwelling, yard, or plot; (2) public taps or standpipes; (3) tube wells or boreholes; (4) protected springs; (5) protected dug wells; and (6) rainwater collection [2]. We streamlined these categories to help students answer this question more easily by modifying the improved water and sanitation definitions of the JMP as follows: a water pipe connection into a dwelling, yard, or plot; a public tap or standpipe; a tube well or borehole; and a protected dug-well or hand-pump for improved water, and a flush or pour-flush toilet or ventilated improved pit toilet for improved sanitation.

### Ethical statement

For this study, ethical approval was obtained from the Federal Ministry of Health, Sudan (FMOH/DGP/RD/TC/2016) and the Korea Association of Health Promotion (130750–20,164 HR-020). Prior to the survey, we provided the survey protocol to the Ministries of Health of all 18 states, including a description of the proposed activities. We explained the survey protocol to the head teachers, teachers, and schoolchildren. Informed consent was obtained from the head teachers and students. Participation in this survey was confidential and entirely voluntary. Withdrawal with no adverse consequences was possible at any time without having to give a reason. If a student agreed to take part, he or she was invited to participate in an interview. Informed consent was obtained from the head teachers of all schools in a written format. A separate informed consent form for students was developed, the script was read by data collectors, and every detail was explained to students point by point. However, it was impractical to obtain written consent from the parents of the schoolchildren due to the large sample size. Instead, schoolteachers informed the parents about the survey details through students and checked for parental consent before launching the survey. Data collection was undertaken using tablet PCs and the data were anonymized. We obtained approval for this procedure from the Institutional Review Board of the Federal Ministry of Health, Sudan. The survey protocol for informed consent complied with the standard procedures of the Federal Ministry of Health, Sudan.

### Sampling

We used two-stage random sampling for the nationwide WASH survey. We applied probability proportional-to-size sampling to select the schools. Twenty students from the second, fourth, and sixth grades were selected at each school. We divided each district into one to three different ecological zones depending on its distance to water bodies (near, less than 1 km; medium, 1–5 km; far, 5 km or more). We defined an ecological zone as an area located within a similar distance from bodies of water in a district. There were only one or two ecological zones in some districts. We used random sampling for schools and students to derive precise estimates of prevalence with a sufficient sample size. Finally, we surveyed 105,167 students from 1,772 primary schools from 390 ecological zones in 183 districts of 18 states across Sudan. The nationwide WASH survey was conducted from December 2016 to March 2017.

### Data collection

In total, 655 people were temporarily employed for the survey, most of whom were government officials or experienced laboratory technicians in state-run hospitals. Participants were interviewed about their behaviors and the water source and type of sanitation used in their household (Supporting Information S1 Text). School-level latrine and water sources were directly observed. We used tablet PCs (SM-Galaxy T350NZAAXAR, Samsung, Seoul, Korea; MediaPad T17.0, Huawei, Shenzhen, China) to enter the laboratory and interview results. The main purpose of using tablet PCs was to help central supervisors conduct real-time monitoring of the survey on a daily basis. State coordinators submitted all the data, which were subsequently exported into SPSS. National coordinators monitored ongoing progress and analyzed the preliminary results on a daily basis. Geographical coordinates were collected by either the PCs when connected to the internet or a handheld GPS device (eTrex, Garmin International, Olathe, KS, USA). We used STATA v.13 (StataCorp LLC, College Station, TX, USA) for statistical analyses in this study. Sample weighting was applied by state according to the sex ratio and population size of each district.

### Indicators measuring heterogeneity and inequality

The percentage of improved water and sanitation of schools or households was estimated at the district and state level. The Theil L and Theil T indices were used to decompose income inequality, and these indices can also be applied to the decomposition of the distribution of access to improved water and sanitation [17,18]. The Gini coefficient is one of the most widely used measures of inequality [19]. We decomposed the inequality across Sudan into within-state, between-state, within-district, and between-state inequalities using the Theil L and Theil T indices. We calculated the Gini coefficient to assess the inequality of access to improved water and sanitation, based on the deviation of the Lorenz curve from the line of perfect equality. It is based on the Lorenz curve, an accumulated frequency curve that compares the distribution of a specific variable with a uniform distribution that represents equality [19]. To calculate Lorenz curves for each state, administrative areas were ranked from smallest to largest by their share of national use; the cumulative proportion of use was then calculated and plotted against the cumulative percentage of population. The greater the deviation of the Lorenz curve from the diagonal line of equal distribution, the greater the inequality. The Gini coefficient was then calculated as twice the area between the diagonal and the Lorenz curve. A Gini coefficient of 1 means total inequality and a coefficient of 0 represents perfect equality. The Gini coefficient is considered comparatively fair if the value is 0.3 or below, values between 0.3 and 0.4 are considered to constitute a warning sign, and considerable inequality is considered to be present if it is greater than 0.4. Coverage of improved water and sanitation ranges from 0 to 1. Pullan and colleagues generated a geographical Gini coefficient, from which they derived a relative geographic inequality (RGI) index to identify relatively unequal states [20]. Outlier districts with higher or lower levels of inequality given their level of coverage were identified using linear regression of the Gini coefficient against national coverage for overall populations, and the RGI score was generated as the difference between the observed and expected Gini coefficient given district coverage based upon this modeled relationship [20]. We investigated whether the Theil indices were associated with the coverage of improved water or sanitation, with the hypothesis that an inverse association would be found.

## Results

**Fig 1:**
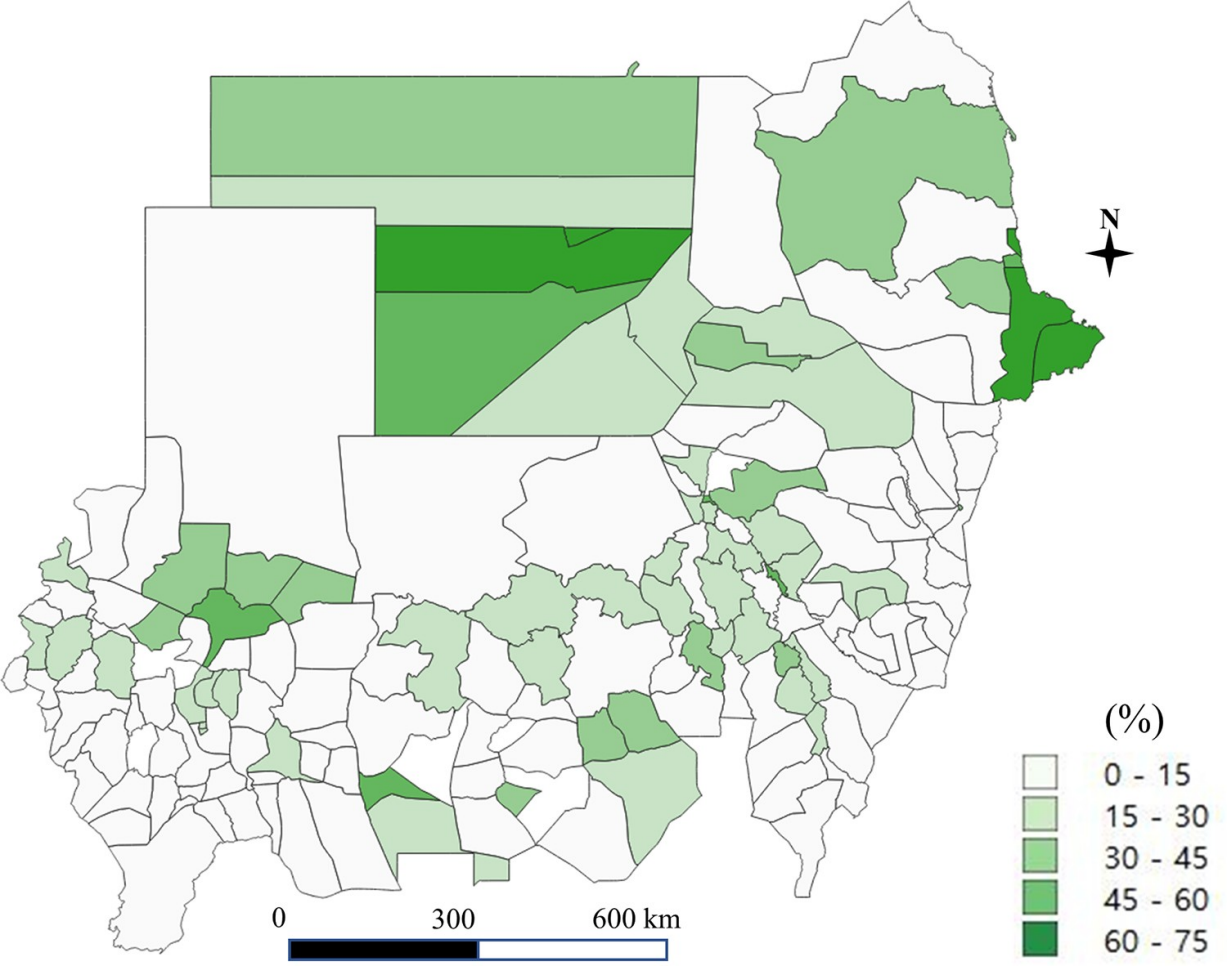
Improved latrine at the household level (the authors generated the map).

**Fig 2:**
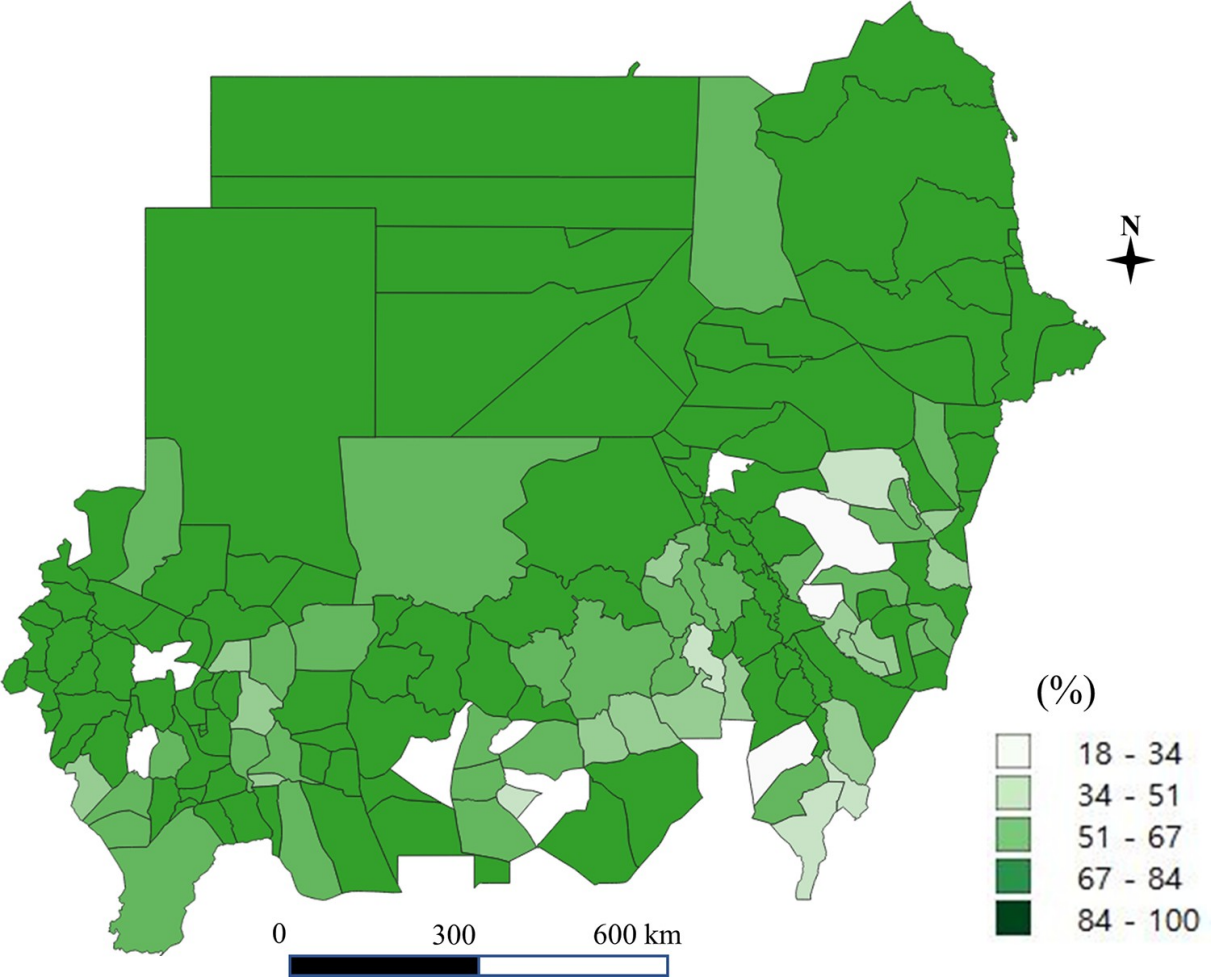
Improved water at the household level (the authors generated the map).

**Fig 3:**
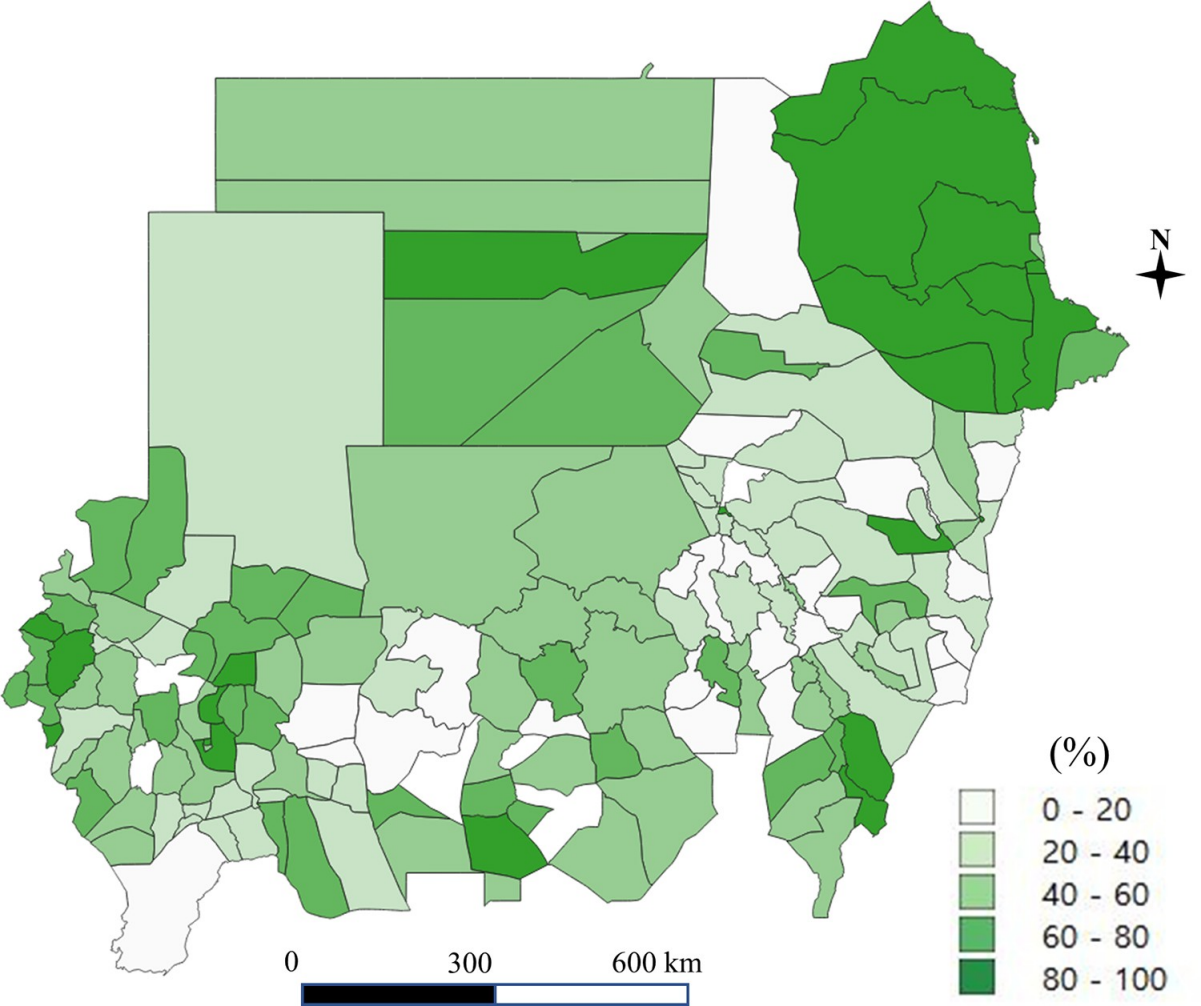
Improved latrine at the school level (the authors generated the map).

**Fig 4:**
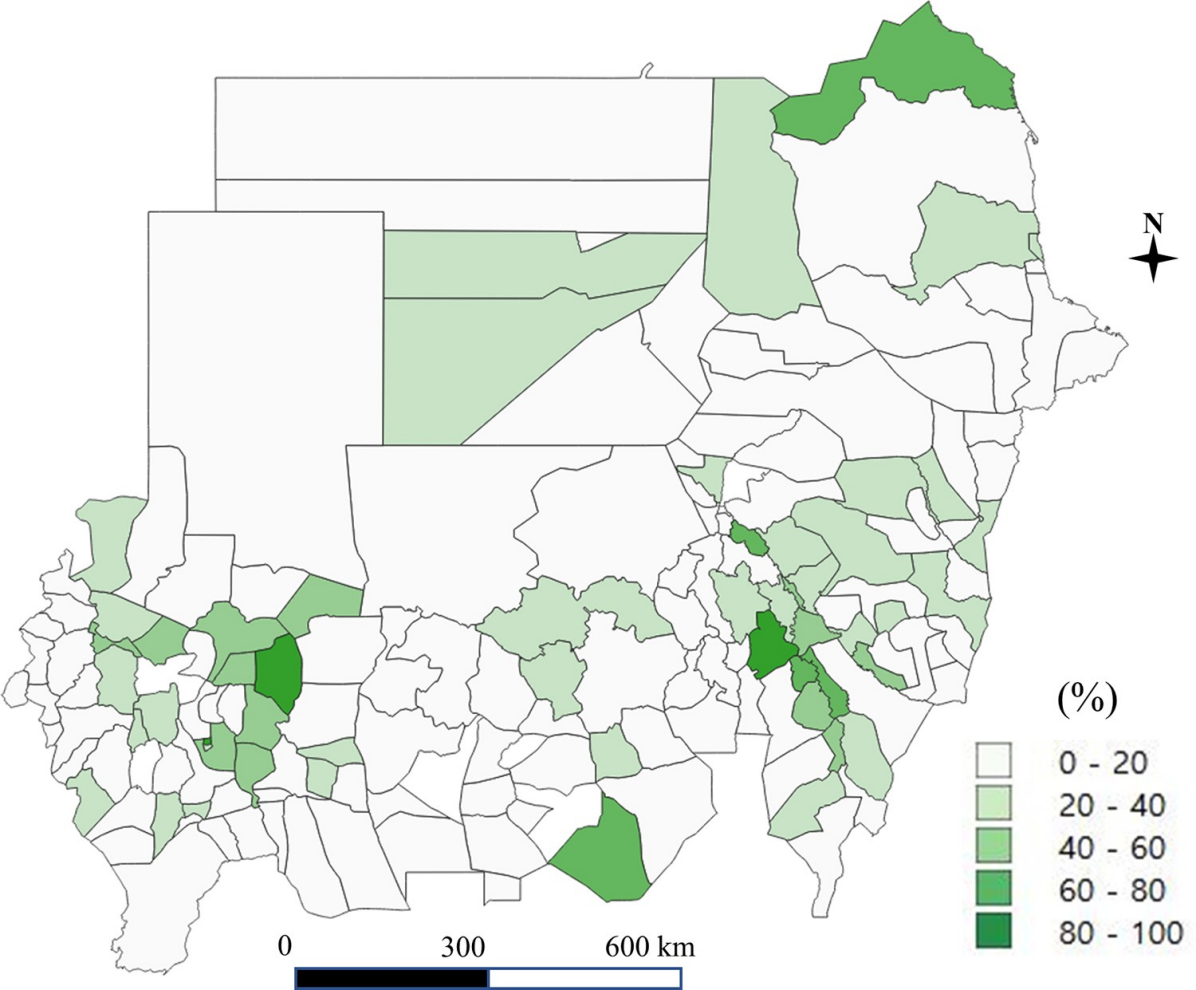
Having water and soap at the school level (the authors generated the map).

A total of 1,711 schools including 108,660 students were obtained from the 2017 nationwide survey in Sudan. The coverage of improved water and sanitation at the household and school level in each district is shown in Figs 1–4. Particularly, Fig 4 shows the percentage of schools with both water and soap. Access to improved drinking water was in the range of 38% to 90%, higher than the percentage of access to improved sanitation, which ranged from 5% to 70% (for details of the coverage of improved water and improved sanitation at the school and household level, see Supporting Information S1 Table).

The national coverage was 86% for improved water and 16% for improved sanitation at the household level (Tables 1 and 2). Low coverage of improved sanitation at the state level was mainly observed in the Darfur states (Central, East and South Darfur), Kassala State, and El Gadaref State, where the proportion of households having access to improved sanitation was below 10%. For improved water, the Blue Nile State and White Nile State had the lowest coverage at the household level.

**Table 1:** Coverage of improved latrines at the household level.

State	Coverage	Theil T	Theil L	Gini	% of within district*
Blue Nile	13.20%	0.570	0.956	0.566	34.6%
Al Gazeira	21.70%	0.165	0.198	0.290	83.0%
Central Darfur	9.50%	0.741	0.710	0.599	74.7%
East Darfur	4.40%	0.975	0.990	0.680	56.3%
El Gadaref	8.60%	0.284	0.446	0.390	81.2%
Al Khartum	31.40%	0.131	0.139	0.285	73.7%
North Kordofan	13.80%	0.213	0.303	0.348	65.8%
Northern	41.40%	0.067	0.068	0.202	82.7%
West Darfur	17.20%	0.094	0.096	0.237	89.2%
West Kordofan	11.80%	0.512	0.670	0.532	59.6%
Kassala	6.10%	0.782	0.973	0.649	63.3%
North Darfur	19.90%	0.492	0.780	0.528	54.0%
Red Sea	36.30%	0.350	0.262	0.420	49.0%
River Nile	14.50%	0.252	0.574	0.357	78.7%
Sinnar	19.50%	0.089	0.099	0.228	82.5%
South Darfur	5.50%	0.643	0.990	0.600	60.0%
South Kordofan	16.90%	0.367	0.668	0.456	73.3%
White Nile	14.90%	0.358	0.540	0.447	72.1%
Overall	15.61%	0.480	0.759	0.529	
Within		0.340	0.616		
Proportion of “within inequality" of “overall “inequality"		71.0%	81.2%		

**Table 2:** Coverage of improved water at the household level.

State	Coverage	Theil T	Theil L	Gini	% of within district*
Blue Nile	60.2%	0.084	0.104	0.215	78.7%
Al Gazeira	89.1%	0.018	0.020	0.089	91.4%
Central Darfur	88.1%	0.011	0.012	0.062	75.1%
East Darfur	83.3%	0.025	0.027	0.123	80.8%
El Gadaref	73.4%	0.088	0.120	0.204	92.2%
Al Khartum	98.2%	0.000	0.000	0.011	64.7%
North Kordofan	76.8%	0.021	0.022	0.112	91.6%
Northern	99.7%	0.000	0.000	0.002	100%
West Darfur	98.1%	0.001	0.001	0.013	91.9%
West Kordofan	90.6%	0.005	0.005	0.053	92.3%
Kassala	80.8%	0.028	0.030	0.128	82.7%
North Darfur	91.2%	0.008	0.008	0.062	81.6%
Red Sea	99.3%	0.000	0.000	0.005	82.7%
River Nile	87.0%	0.005	0.006	0.051	95.5%
Sinnar	99.4%	0.000	0.000	0.005	81.8%
South Darfur	87.7%	0.018	0.023	0.079	79.7%
South Kordofan	81.5%	0.018	0.019	0.101	88.7%
White Nile	65.5%	0.024	0.026	0.116	98.1%
Overall	85.8%	0.025	0.032	0.104	
Within		0.017	0.024		
Proportion of "within inequality" of "overall inequality"		68.1%	75.0%		

We discovered that access to improved water or sanitation was greatly unequal even within states. For example, we estimated that improved drinking water was 18% in the Al-Tadamon district in the Blue Nile state, while the Al-Damazein district in the same state had 89% access, (Supporting Information S1 Table). A high disparity within states was also observed with regard to improved sanitation, with the Tokar district in the Red Sea State having 75% coverage, whereas the Durdaib district in the same state had 2% coverage.

Geographical heterogeneity was prominent in the Central Darfur, South Darfur, East Darfur, Kassala, West Kordofan, and Blue Nile States, all of which showed severe inequality in access to an improved latrine at the household level in terms of the Theil T or Theil L index. Concerning improved sanitation, coverage varied from less than 10% for 85 districts to 50% for 10 districts (Supporting Information S1 Table).

The availability of improved water in 10 districts was less than 50% despite the national coverage of 86%. We found many outliers in both improved water and sanitation indicators. For example, the Kassala district in Kassala State had 36% coverage of improved latrines at the household level, while there were no students whose households had an improved latrine in four districts in the same state.

**Fig 5:**
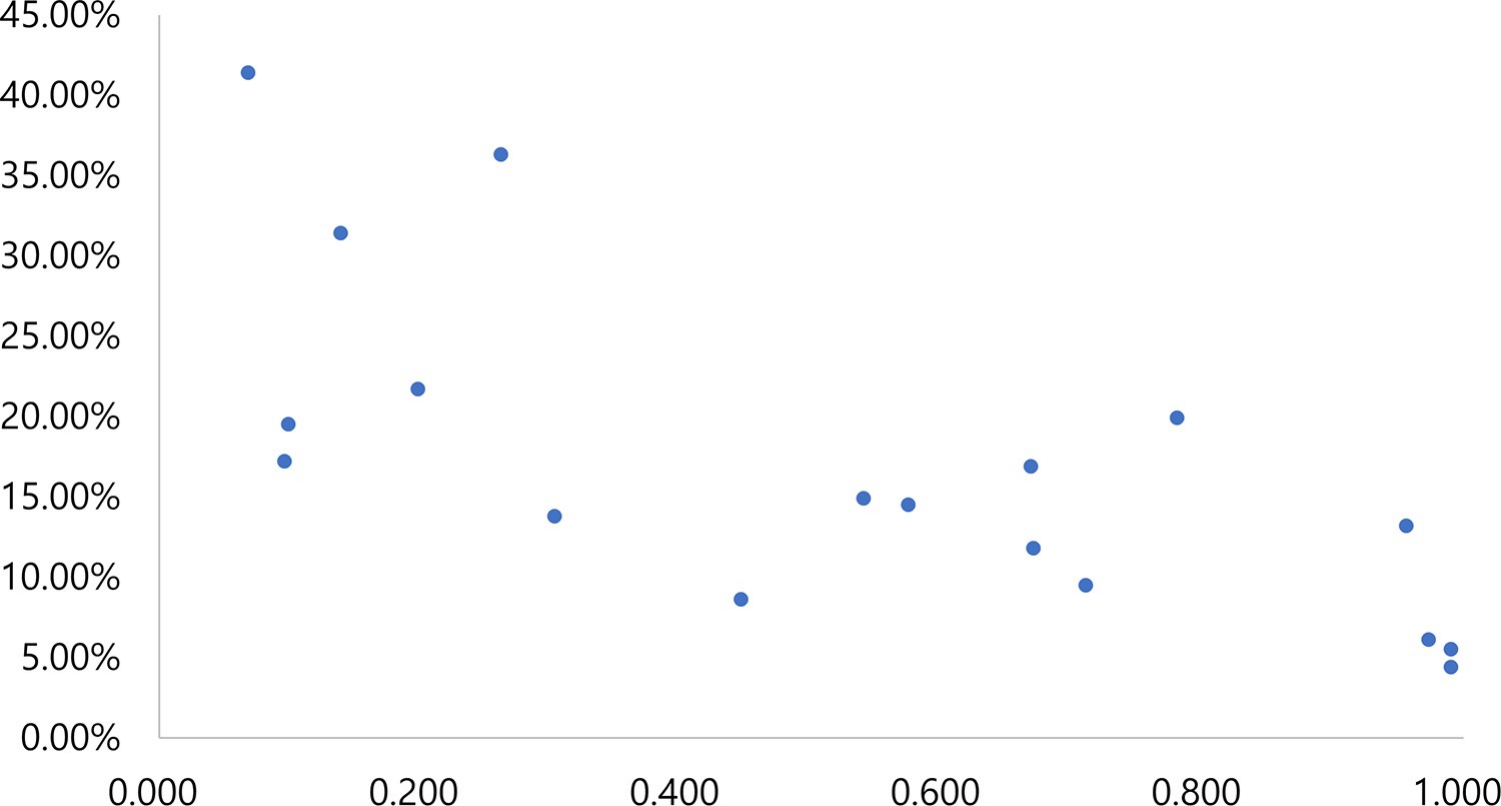
Correlation between Theil T index and coverage of an improved household latrine (x-axis: Theil T index; y-axis: Coverage, the authors generated the map).

**Fig 6:**
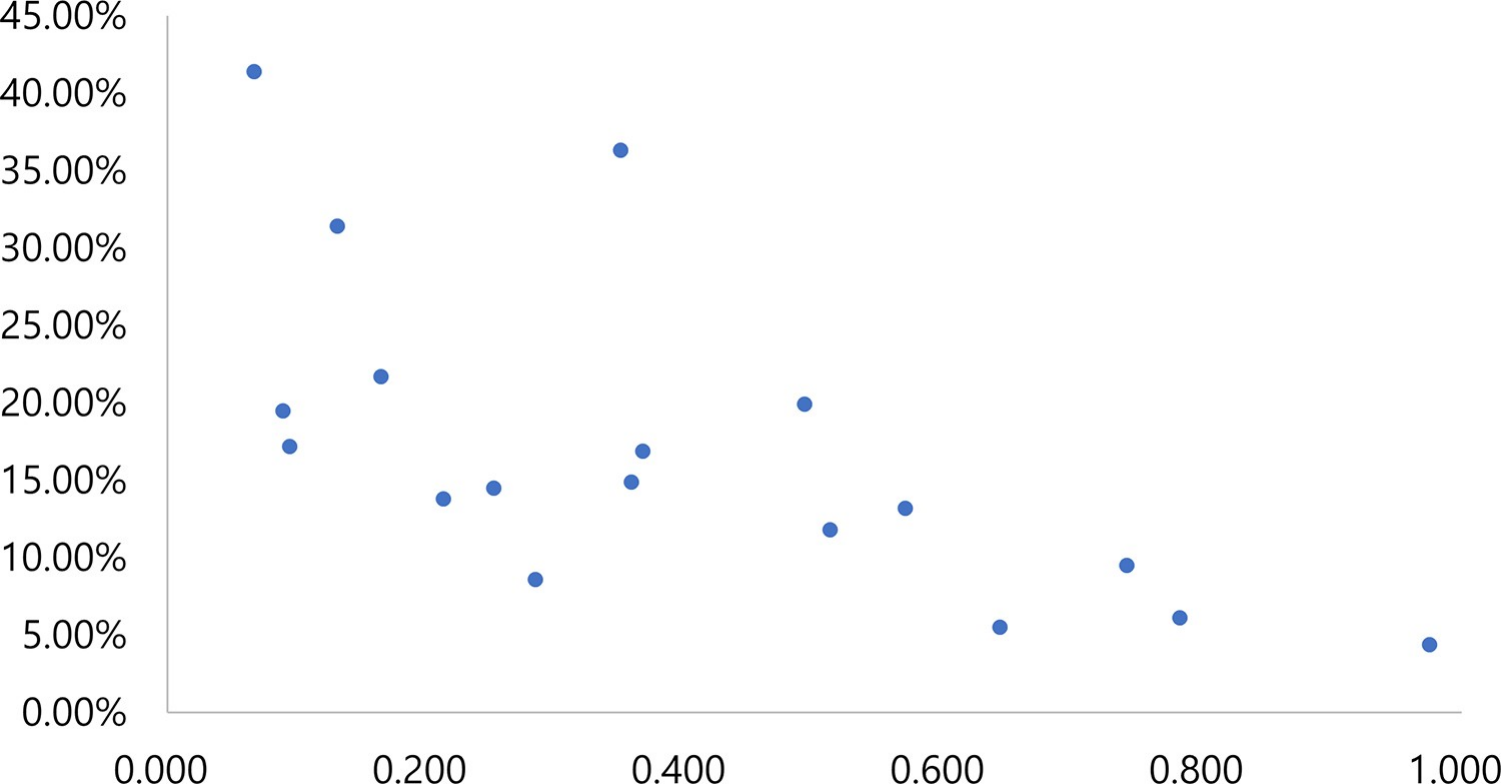
Correlation between Theil L index and coverage of an improved household latrine (x-axis: Theil L index; y-axis: Coverage, the authors generated the map).

The overall inequality in the coverage of improved sanitation went beyond the warning limit of 0.4 for the Gini coefficient (Table 1). Tables 1–4 decompose the Theil L and Theil T indices, as well as the Gini coefficient, presenting within- and between-state inequality. First, the inequality in terms of the Theil L and Theil T indices, as well as the Gini coefficient, was always higher for improved sanitation than for improved water at the household level. Second, within-state inequality accounted for 66% or more of national inequalities in the distribution of improved sanitation and drinking water for both the Theil L and Theil T indices, which always showed inverse correlations with both improved sanitation and improved drinking water (Figs 5 and 6). Table 5 lists the key 30 districts with greater inequality than expected.

**Table 3:** Coverage of improved latrines at the school level.

State	Coverage	Theil T	Theil L
Blue Nile	69.1%	0.021	0.023
Al Gazeira	22.7%	0.139	0.154
Central Darfur	46.0%	0.026	0.028
East Darfur	42.0%	0.054	0.054
El Gadaref	29.3%	0.095	0.109
Al Khartum	49.5%	0.099	0.090
North Kordofan	51.8%	0.057	0.065
Northern	59.9%	0.017	0.016
West Darfur	77.7%	0.015	0.015
West Kordofan	48.0%	0.100	0.127
Kassala	38.1%	0.128	0.131
North Darfur	52.0%	0.081	0.087
Red Sea	91.9%	0.014	0.015
River Nile	26.9%	0.166	0.151
Sinnar	35.2%	0.138	0.156
South Darfur	44.9%	0.088	0.095
South Kordofan	44.3%	0.167	0.198
White Nile	31.6%	0.219	0.301
Overall	47.2%	0.123	0.145
Within		0.080	0.104
Proportion of "within inequality" of "overall inequality"		65.0%	71.7%

**Table 4:** Coverage of improved water at the school level.

State	Coverage	Theil T	Theil L
Blue Nile	61.8%	0.118	0.137
Al Gazeira	61.4%	0.032	0.033
Central Darfur	42.3%	0.061	0.065
East Darfur	32.5%	0.112	0.128
El Gadaref	47.1%	0.114	0.125
Al Khartum	93.8%	0.002	0.002
North Kordofan	42.9%	0.079	0.095
Northern	100.0%	0.000	0.000
West Darfur	43.1%	0.101	0.119
West Kordofan	21.1%	0.080	0.085
Kassala	66.7%	0.065	0.078
North Darfur	21.1%	0.147	0.125
Red Sea	39.7%	0.201	0.229
River Nile	81.7%	0.022	0.023
Sinnar	67.3%	0.033	0.036
South Darfur	27.6%	0.174	0.205
South Kordofan	60.6%	0.088	0.101
White Nile	40.5%	0.118	0.135
Overall	48.0%	0.139	0.164
Within		0.078	0.103
Proportion of "within inequality" of "overall inequality"		56.1%	62.8%

**Table 5:** Key districts with high RGI scores that had higher GINI values observed than expected.

State	District	Coverage of imp_Latrine (%)	Gini_latrine	RGI Score^a^	State	District	Coverage of imp_Water (%)	Gini_water	RGI Score^a^
El Gadaref	Alfashagah	13%	0.681	0.336	South Kordofan	Gadeir	89%	0.326	0.261
North Darfur	Almalhah	12%	0.678	0.333	Blue Nile	Geissan	47%	0.445	0.188
Al Gazeira	Alkamlein	14%	0.637	0.291	South Darfur	Kutom	33%	0.503	0.183
North Kordofan	Barah	19%	0.630	0.282	West Nile	Rubak	93%	0.212	0.161
Al Gazeira	Algorashi	4%	0.621	0.279	South Kordofan	Algooz	91%	0.213	0.153
Central Darfur	Zalingai	20%	0.627	0.278	Blue Nile	AlRosaeris	63%	0.337	0.153
West Darfur	Jabal moon	8%	0.620	0.277	South Kordofan	Abo Jebaiha	96%	0.173	0.137
Al Khartoum	Khalawei	16%	0.603	0.256	North Darfur	Kalamendow	68%	0.286	0.124
Central Darfur	Makjur	3%	0.594	0.253	East Darfur	Yasein	69%	0.277	0.121
Central Darfur	Bundasi	6%	0.592	0.249	El Gadaref	Galaa Alnahel	65%	0.285	0.108
South Kordofan	Habeila	0%	0.589	0.249	South Darfur	Rehaid Albrdei	68%	0.255	0.093
Sinnar	Aldali	13%	0.592	0.247	El Gadaref	Albutanah	18%	0.483	0.093
River Nile	Burbur	18%	0.594	0.246	El Gadaref	Elgreaishah	80%	0.193	0.086
North Kordofan	Um dam Haj Ahmed	12%	0.590	0.245	North Darfur	Dar Elsalam	64%	0.263	0.083
West Kordofan	Wadbanda	7%	0.579	0.236	Kassala	West Kassala	54%	0.305	0.077
Sinnar	East Sinnar	9%	0.573	0.229	West Nile	Aldewaim	81%	0.174	0.072
Al Gazeira	South Algazeira	14%	0.567	0.221	South Darfur	Eid Elfursan	82%	0.166	0.068
West Darfur	Baidah	14%	0.563	0.217	El Gadaref	East Algdareif	82%	0.164	0.066
West Kordofan	Alkhwai	4%	0.556	0.214	South Darfur	Um Dafoog	73%	0.205	0.066
West Nile	Tandalti	10%	0.556	0.211	South Kordofan	Alleiry	100%	0.082	0.064
South Kordofan	Abo Kursholah	19%	0.555	0.207	Kassala	Rifi Atbrah	50%	0.311	0.064
South Darfur	Rehaid Albrdei	6%	0.548	0.205	East Darfur	Asalaiah	52%	0.302	0.064
South Darfur	Tuls	4%	0.544	0.202	East Darfur	Eldeain	83%	0.156	0.063
East Darfur	Abo Karinka	3%	0.542	0.201	River Nile	Abo Hamed	83%	0.155	0.062
North Kordofan	Shikan	21%	0.547	0.198	River Nile	Burbur	86%	0.141	0.061
Central Darfur	Wadi Salih	8%	0.540	0.197	River Nile	Atbarah	84%	0.150	0.058
West Nile	Aljabalain	13%	0.540	0.195	West Kordofan	Kaileik	84%	0.147	0.055
West Kordofan	Aldibab	13%	0.534	0.188	River Nile	Amatamah	86%	0.137	0.055
West Darfur	Kulbos	16%	0.535	0.188	West Nile	Galy	65%	0.234	0.055
El Gadaref	Center Algdareif	21%	0.537	0.187	River Nile	Aldamar	86%	0.133	0.054

^a^ The RGI (relative geographic inequality) score measures relative inequality when given coverage levels.

Negative values indicate a lower than expected inequality, while positive values indicate greater than expected inequality All the values of the RGI score in this table are significantly different from 0.

## Discussion

In this study, we analyzed and mapped the spatial heterogeneity and inequality of access to improved water and sanitation at the household and school levels in Sudan. The states with the lowest coverage and highest degree of inequality were identified, such as East Darfur and Kassala States in improved sanitation at the household level and River Nile State at the school level. The geographical inequality of improved sanitation was more profound than that of improved drinking water in Sudan, which is consistent with previous studies in other countries [21,22]. There was substantial geographical heterogeneity of improved sanitation across the nation between states and districts.

It is already known that WASH coverage differs among geographic areas. Several studies have explored WASH coverage in Sudan, but most were undertaken in only a few districts or states [23–26]. UNICEF recently assessed WASH coverage at the national and state level, but not at the district level [27]. There has been no analysis of the sub-national geographical distribution of WASH services at the district level in Sudan. While the previous nationwide survey by UNICEF presented national or state-level coverage in WASH, we designed this study to derive district-level representative data with an appropriate sample size and sampling method [7]. This study clearly illustrates that WASH coverage varies substantially even within a given state, which helps to highlight the importance of providing WASH coverage for smaller geographical areas than the state level.

We do not intend to replace the existing information on WASH coverage across Sudan developed by UNICEF, but to provide contemporary maps at the district level and to provide information on inequalities in WASH by quantifying their magnitude with data that can be used for adequate investment and planning by the government and other stakeholders in the global health and development communities. In addition, the definition of improved sanitation used herein is different from that of the JMP [2]. We used a more stringent definition reflecting the argument that sanitation infrastructure could become a source of disease transmission if poorly maintained or constructed. Therefore, the coverage in this study is not directly comparable with the results reported by the JMP.

A previous study suggested that countries with greater inequality in access to improved water experienced more severe inequality in access to improved sanitation [20]. The authors argued that marginalized people and out-of-reach areas suffer the compounded effect of unimproved water and sanitation, as well as inadequate hygiene services. In this study, we did not find such a pattern at the household or school level.

Countries willing to increase WASH coverage should make efforts to ensure the adequate targeting of investments and overcome the patchy implementation of WASH interventions, which requires careful planning. Formulating strategies for reaching out to people in the lowest WASH coverage areas should not be simply rhetoric, but should be deliberately adopted by the government. The global health community should also be responsible for providing these areas with more investment or assistance to improve WASH coverage and reduce inequality.

Furthermore, it would be helpful to understand inequalities in WASH coverage in order to develop adequate strategies to prevent and control many infectious diseases [28–34]. For instance, people without access to adequate WASH services are prone to some NTDs like schistosomiasis, trachoma, and soil-transmitted helminthiasis [28–32]. Understanding the geographic distribution of WASH will also provide insights into the epidemiology of other infectious diseases including cholera, typhoid, and the like [33,34]. We conducted this study to help the Sudanese government better identify hotspot areas of some neglected tropical diseases (NTD), including schistosomiasis and soil-transmitted helminthiasis, so that they could carry out intensive and integrated NTD control and elimination activities. Accessibility of WASH services is critical for elimination strategies in combination with mass drug administration (MDA) [35,36]. There are growing demands for concerted efforts to develop intersectoral interventions, collaboration, and coordination between WASH and the NTD arena [37,38]. Particularly, Sudan is now transitioning from control to elimination of schistosomiasis [36,39]. To achieve this goal, approaches and strategies should be changed to effectively control and prevent schistosomiasis. In this regard, improvements in WASH both at the household and school levels are one of the key components for moving from control towards elimination of schistosomiasis [35]. Existing WHO guidelines on MDA for schistosomiasis were primarily developed for the purpose of control, not elimination; thus, some countries, including Sudan, are struggling to transition from control to elimination through the interruption of schistosomiasis transmission [35,39]. We believe that this study can be a useful tool for formulating strategies to eliminate schistosomiasis in Sudan because these strategies could help accurately target the districts most in need in terms of WASH coverage by combining WASH investments with MDA to interrupt the transmission of schistosomiasis. We suggest that the districts with lowest coverage of improved water and sanitation and a high inequality index are potential candidates as target areas for nationwide WASH and/or NTD control and elimination programs.

WASH in schools provides a range of benefits such as reducing absenteeism among girls, enhancing educational performance, and improving child health. SDGs incorporate universal access to WASH in schools [40]. Universal access encompasses schools and health facilities, not only households [41]. Correspondingly, the global indicators of the SDGs include the proportion of schools with access to basic drinking water, single-sex basic sanitation, and basic handwashing facilities [41]. Despite the benefits of WASH and its incorporation into global goals, WASH services in schools remain poor and are not adequately monitored in many countries, including Sudan.

The WHO/UNICEF JMP generated baseline reports on WASH in schools in 2018 and updated them in 2020 to track progress [42,43]. However, WASH in schools in Sudan was not reported even in the 2020 JMP findings because of the lack of a routine data information system on WASH in schools [43]. Recently, UNICEF Sudan published a report on WASH in schools from 54 districts, which presented national and state coverage [27]. As we stated with regards to the UNICEF report on household-level data, this study based on data collection from 1,772 schools of 183 districts across Sudan can also serve as a complementary source of information on contemporary WASH coverage at the district level. In addition, our study provides further information about inequalities in WASH in schools, which were not analyzed in the UNICEF report.

The global health crisis due to COVID-19 has underscored the importance of WASH in schools for interrupting the transmission of infection and protecting health [43,44]. In response to the COVID-19 pandemic, there have been global school closures since early 2020. Concerns have emerged in the global community that these prolonged school closures will present unprecedented risks to children in many aspects such as educational outcomes and disruption of school-based services like welfare, protection, and nutrition [45]. To identify various measures to reopen schools, the WHO and UNICEF developed a guideline to prevent and control infectious diseases including COVID-19 [46]. They re-emphasized the critical importance of WASH in schools and recommended implementing safe water, improved sanitation, and adequate hygiene services in schools. Correspondingly, many governments started to formulate strategies to operate schools during the pandemic by incorporating WASH improvements in schools [43]. In response to this global trend, several countries launched assessments of WASH in schools. For instance, Ecuador undertook a nationwide survey on WASH services in school in 2020 and produced a nationwide map of the geographical distribution of WASH in schools, and the country has been calling for more support from the global community based on that assessment [43]. Still, many countries have not conducted a nationwide assessment of WASH services in schools. Thus, this study will be useful for understanding the distribution of WASH services in schools at the national, state, and district levels, and will also be relevant as a baseline for monitoring progress in future surveys. We expect the Sudanese government to establish a regular monitoring mechanism to identify the status of WASH in schools and track its progress.

Consistent with previous studies, this study found that inequality decreased as coverage of improved water and sanitation increased [20,22]. In addition, within-district and within-state inequality was greater than between-district and between-state inequality, unlike results from other countries [22]. In contrast with the results reported from Nepal, administrative and governance differences between districts might not the factor most strongly influencing inequality in water and sanitation coverage [22]. Rather, we may argue that the inequality in water and sanitation access is a persistent challenge found across the nation. For this reason, we appeal to the Federal Ministry of Health and 18 State Ministries of Health to pay close attention and make efforts to resolve this issue collectively.

A range of national strategic frameworks, plans, and roadmaps for improvement in WASH have been developed in recent years [13,47,48]. For instance, the National Sanitation Strategic Framework set directions to move forward with the SDGs [47]. The Sudanese government and UNICEF Sudan jointly developed the National Roadmap to End Open Defecation in Sudan and they considered making Sudan open-defecation-free by 2022 a vital milestone to reach the goal of universal access to basic sanitation services [13]. However, the ineffective utilization of available funding and inadequate or outdated data on WASH are considered some of the key challenges [12]. Institutional arrangements for WASH sector coordination are urgently needed since there is no active development and emergency sector coordination mechanism in Sudan [12]. We appeal to the Sudanese government and WASH community to start discussions on developing a WASH sector coordination forum encompassing the development and emergency sector. We hope that this study can be used as complementary information to help the Sudanese government and its development partners develop adequate targeting of resources and to monitor the progress of such initiatives. While appealing to policy makers to improve WASH in schools, there are many opportunities for front-liners to take relevant steps at the school level. Establishing health clubs and activating parent-teacher associations have been found to have a major impact on improving WASH in schools [49,50]. Strong school leadership is also another key factor [49,50].

When identifying types of water, sanitation, and handwashing facilities, we made direct observations of each facility to avoid any possible reporting bias. However, for household-level WASH facilities, we had to rely on students’ reports because it was almost impossible to physically visit all the households of 105,167 students. Although we simplified the number of answers per question to avoid any possible confusion, a limitation of this study is that the validity of the students’ reports remained unchecked. We used the Gini coefficient to measure the magnitude of inequality, since it is a widely used assessment method that is helpful for comparing the results with other studies [19]. However, it is not possible to decompose the inequality into between-group and within-group inequality using the Gini coefficient. To measure the extent of inequality between and within states/districts, we used the Theil T and the Theil L indices [17,18]. The Theil T index was used as a complementary index to the Theil L index when there was an instance of zero population in a unit [17,18].

To our knowledge, this is the first study to measure geographical heterogeneity and inequalities in improved water and sanitation coverage across Sudan. The study may help to prioritize resource allocation to areas with the greatest water and sanitation needs. We detected substantial geographical heterogeneity and inequality even within districts across Sudan, distinct from the patterns observed in national statistics. The results showed that the main inequality was found within states and within districts. The inequality in coverage of improved sanitation and water needs to be tackled as part of the district health policy, and the Federal Ministry of Sudan must address this issue, since it is a basic human right. The most vulnerable groups need to be prioritized to accelerate the achievement of universal coverage of improved water and sanitation.

## Supporting information

S1 Table Water, sanitation and hygiene coverage by district in Sudan. (XLSX)

S1 Text Questionnaire. (PDF)
