# Supplementary material for: Unequal geographic distribution of water and sanitation at the household and school level in Sudan
Source: PLoS One. 2021 Oct 15;16(10):e0258418. doi: 10.1371/journal.pone.0258418 (PMC8519438; doi:10.1371/journal.pone.0258418)
Supplement: S1 Text — (PDF) [file pone.0258418.s002.pdf]

S1. Text questionnaire set

Questionnaire for students

State [|] Locality [|] Ecological Zone [ ] School [ ] Student [|]

1. Age [ ]

2. Sex ① boy ② girl

3. Parents' occupation [ ]

4. What types of water source do you for drinking at home?

   ① protected (boreholes, hand-pumps, protected spring, tap or standpipe (public/on premise))

   ② unprotected (river, stream, pond, unprotected spring)

5. Do you contact water bodies (river, stream, lake, irrigation canal, reservoir) more than two times a week?

   ① Yes ② No

6. Why do you contact water bodies?

   ① Fetching water ② bathing ③ laundry ③ playing (swimming) ④ for livestock

   ⑤ Others(_________________)

7. Do you have latrine in your household compound?

   ① I don't have any latrine ② Simple pit latrine

   ③ ventilated improved pit latrine ④ (pour) flush toilets/siphon toilet ⑤ others

8. Where do you defecate? ① Latrine ② open defecate

Observation list/questionnaire for a school (head teacher)

State ☐☐ Locality ☐☐ Ecological Zone ☐ School ☐

1. Geographic location of the school: [observation]
2. What types of water source does the school have? [observation]

   ① protected (boreholes, hand-pumps, protected spring, tap or standpipe)

   ② unprotected (river, stream, pond, unprotected spring)
3. Does the school have latrine school compound? [observation]

   ② It doesn't have any latrine ② Simple pit latrine

   ③ ventilated improved pit latrine ④ (pour) flush toilets/siphon toilet ⑤ others
4. Please describe all the surrounding villages where students live.
5. Does the school have any curriculum to regularly teach Bilharzia and STH?

   ①Yes ② No
6. Has the school had any special health program, campaign such as nutrition, vaccination or any other? Please describe it if there has been.

   ____
7. When was last time when MDA intervention was conducted?

   (Month: Year:)
